# Draft genome sequences of two *Halobacteriovorax* strains isolated from Apalachicola Bay, Florida

**DOI:** 10.1128/mra.00851-25

**Published:** 2025-12-22

**Authors:** Lauren Speare, Macey N. Coppinger, Grisel Fierros-Romero, Henry N. Williams

**Affiliations:** 1 School of Biological Sciences and Center for Microbial Dynamics and Infection, Georgia Institute of Technology 1372, Atlanta, Georgia, USA; 2 School of the Environment, Florida Agricultural and Mechanical University 7822, Tallahassee, Florida, USA; University of Southern California, Los Angeles, California, USA

**Keywords:** BALO, marine microbiology, predatory bacteria

## Abstract

Predatory bacteria are common in aquatic environments and associated with eukaryotic hosts. Here, we report draft genome sequences for two marine isolates, *Halobacteriovorax* sp. strains GFR7 and GFR8, that were isolated from Dickerson Bay in Apalachicola Bay, Florida, USA.

## ANNOUNCEMENT

*Halobacterivorax* (formerly *Bacteriovorax*) are obligate bacterial predators within marine and brackish habitats (1–5). Taxa within this genus are considered “keystone species” due to their predicted top-down structuring role (1).

Here, we introduce the genomes of two *Halobacteriovorax* strains, GFR7 and GFR8, that were isolated from Dickerson Bay in Apalachicola Bay, Florida, USA near the Gulf Specimen Marine Laboratory (30.025614°N, 84.382839°W). Water samples were collected, stored on ice, and transported to Florida A&M University. Strains were isolated from PP20 agar plaques on *Vibrio vulnificus* prey. To acquire genomic DNA, plaques were generated and resuspended in 40 mL of freshly grown *V. parahaemolyticus* culture. Once cleared, cultures were filtered twice through 0.45 µm syringe filters. Filtrates were centrifuged at 21,300 × *g* for 20 min to pellet, and then DNA was extracted with an Invitrogen PureLink Genomic DNA Kit according to manufacturer instructions (Thermo Fisher Scientific). DNA quantity and quality were determined using a Nanodrop spectrophotometer. gDNA samples were then sent to SeqCenter, LLC (Pittsburgh, PA) for Illumina whole-genome sequencing using the Illumina DNA Prep Kit. Sequencing was performed on an Illumina NovaSeq X Plus sequencer and resulted in paired-end 150 bp reads: 2,926,628 and 3,073,590 paired reads for GFR7 and GFR8, respectively (Table 1). Default parameters were used for all software, unless otherwise specified. Raw reads had a Phred Q30 score of 95.5% for GFR7 and 94.7% for GFR8 (6). Raw reads were assembled into contigs using Unicycler (7) on the Galaxy (8) (usegalaxy.org version 25.1.rc1) public server. Contigs were uploaded into Geneious Prime 2025.0.3, extended, and combined using the default map to reference and *de novo* assembly actions. Genomes were annotated via the Prokka Pipeline (9, 10) on Galaxy (8); upon upload to National Center for Biotechnology Information (NCBI), data were reanalyzed using the NCBI Prokaryotic Genome Annotation Pipeline (PGAP) (11).

**TABLE 1:** Sequencing statistics for GFR7 and GFR8^*a*^

Strain	Total reads	Genome size (bp)	N50 (bp)	Avg. coverage	G/C content	Coding regions	Putative proteins
GFR7	2,926,628	3,420,318	2,950,718	120x	37%	3,318	3,272
GFR8	3,073,590	3,420,369	2,950,716	125x	37%	3,320	3,274

Individual contig information							
	Contig 1 accession	Contig 1 size (bp); circularized	Contig 1 size (bp); circularized	Contig 2 accession	Contig 2 size (bp)	Contig 3 accession	Contig 3 size (bp)
GFR7	JBPBLS010000001.1	97,014	97,014	JBPBLS010000002.1	372,586	JBPBLS010000003.1	2,950,718
GFR8	JBPBLR010000001.1	97,014	97,014	JBPBLR010000003.1	372,639	JBPBLR010000002.1	2,950,716

^*a*^ Contigs are shown ordered smallest to largest.

The final draft genomes of GFR7 and GFR8 are 3,420,318 and 3,420,369 bp long, respectively, in three contigs, have 120× and 125× genome coverage, an N50 of with 2,950,718 and 2,950,716 bp, respectively, and a G+C content of 37% for each strain (Table 1). We determined that contig 1 was a closed plasmid via the Unicycler Assembler (7) on Galaxy (8) manually and using the map to reference feature by checking that the overhanging mapped reads from each contig end matched one another. A total of 3,318 and 3,320 DNA coding regions were identified, including 3,272 and 3,274 encoding putative proteins and 38 encoding RNAs, respectively, for GFR7 and GFR8. We predicted functions for 2,103 for GFR7 and 2,105 for GFR8 of these proteins, while the other 1,207 proteins were assigned as hypothetical for both strains using Prokka on Galaxy (8). RefSeq Masher (12) determined that the most closely related genome on NCBI was *Bacteriovorax* sp. BAL6_X (PRJNA210328), with 91.5% ANI to GFR7 and GFR8, suggesting these strains are in the same genus. BAL6_X was deposited in NCBI in 2013, after which the *Bacteriovorax* genus was reclassified as *Halobacteriovorax* (3).

## Data Availability

These genome sequences of GRF7 and GRF8 are available in GenBank under accession no. SAMN48918368 and SAMN48918369, respectively, in the BioProject number PRJNA1272625. Cultures of GFR7 and GFR8 are available upon request.

## References

[1] Williams HN, Lymperopoulou DS, Athar R, Chauhan A, Dickerson TL, Chen H, Laws E, Berhane T-K, Flowers AR, Bradley N, Young S, Blackwood D, Murray J, Mustapha O, Blackwell C, Tung Y, Noble RT. 2016. Halobacteriovorax, an underestimated predator on bacteria: potential impact relative to viruses on bacterial mortality. ISME J 10:491–499. doi:10.1038/ismej.2015.129 26251870 PMC4737939

[2] Rice TD, Williams HN, Turng BF. 1998. Susceptibility of bacteria in estuarine environments to autochthonous bdellovibrios. Microb Ecol 35:256–264. doi:10.1007/s002489900081 9569283

[3] Koval SF, Williams HN, Stine OC. 2015. Reclassification of Bacteriovorax marinus as Halobacteriovorax marinus gen. nov., comb. nov. and Bacteriovorax litoralis as Halobacteriovorax litoralis comb. nov.; description of Halobacteriovoraceae fam. nov. in the class Deltaproteobacteria. Int J Syst Evol Microbiol 65:593–597. doi:10.1099/ijs.0.070201-0 25406234 PMC4811658

[4] Ye X-S, Chen M-X, Li H-Y, He X-Y, Zhao Y. 2019. Halobacteriovorax vibrionivorans sp. nov., a novel prokaryotic predator isolated from coastal seawater of China. Int J Syst Evol Microbiol 69:3917–3923. doi:10.1099/ijsem.0.003703 31498060

[5] Enos BG, Anthony MK, DeGiorgis JA, Williams LE. 2018. Prey range and genome evolution of Halobacteriovorax marinus predatory bacteria from an estuary. mSphere 3:e00508-17. doi:10.1128/mSphere.00508-17 PMC5760749 29359184

[6] Andrews S. 2025. FastQC: a quality control tool for high throughput sequence data

[7] Wick RR, Judd LM, Gorrie CL, Holt KE. 2017. Unicycler: resolving bacterial genome assemblies from short and long sequencing reads. PLoS Comput Biol 13:e1005595. doi:10.1371/journal.pcbi.1005595 28594827 PMC5481147

[8] Abueg LAL, Afgan E, Allart O, Awan AH, Bacon WA, Baker D, Bassetti M, Batut B, Bernt M, Blankenberg D, et al.. 2024. The Galaxy platform for accessible, reproducible, and collaborative data analyses: 2024 update. Nucleic Acids Res 52:W83–W94. doi:10.1093/nar/gkae410 38769056 PMC11223835

[9] Cuccuru G, Orsini M, Pinna A, Sbardellati A, Soranzo N, Travaglione A, Uva P, Zanetti G, Fotia G. 2014. Orione, a web-based framework for NGS analysis in microbiology. Bioinformatics 30:1928–1929. doi:10.1093/bioinformatics/btu135 24618473 PMC4071203

[10] Seemann T. 2014. Prokka: rapid prokaryotic genome annotation. Bioinformatics 30:2068–2069. doi:10.1093/bioinformatics/btu153 24642063

[11] Tatusova T, DiCuccio M, Badretdin A, Chetvernin V, Nawrocki EP, Zaslavsky L, Lomsadze A, Pruitt KD, Borodovsky M, Ostell J. 2016. NCBI prokaryotic genome annotation pipeline. Nucleic Acids Res 44:6614–6624. doi:10.1093/nar/gkw569 27342282 PMC5001611

[12] Ondov BD, Treangen TJ, Melsted P, Mallonee AB, Bergman NH, Koren S, Phillippy AM. 2016. Mash: fast genome and metagenome distance estimation using MinHash. Genome Biol 17:132. doi:10.1186/s13059-016-0997-x 27323842 PMC4915045

